# Management of breech presentation with a large pelvic hydatid cyst in late pregnancy in Tibet: a case report

**DOI:** 10.1186/s12884-022-05180-2

**Published:** 2022-11-20

**Authors:** Ping Li, Yang Wang, Qi Yang, Ma Ni, Jianhui Fan, Zeping Huang

**Affiliations:** 1 Department of Obstetrics and Gynecology, the Third Affiliated Hospital of Sun Yat-Sen University, Guangzhou, China; 2 Laboratory of Biochemistry, the Third Affiliated Hospital of Sun Yat-Sen University, Guangzhou, China; 3 Department of Pharmacy, the Third Affiliated Hospital of Sun Yat-Sen University, Guangzhou, China; 4 Department of Obstetrics and Gynecology, Chaya County People’s Hospital, Tibet, China; 5 Department of Ultrasonography, the Third Affiliated Hospital of Sun Yat-Sen University, No. 600, Tianhe road, 510630 Guangzhou, China

**Keywords:** Hydatid cystic disease, Pregnancy, Management, Tibet

## Abstract

**Background:**

Hydatid cystic disease (HCD) is primarily a disease of sheep and cattle. Human beings are accidental hosts. It is prevalent in the Tibet Autonomous Region (TAR) of China. In pregnancy, it can cause many complications.

**Case presentation:**

We present a multigravida with breech presentation at 37 weeks of pregnancy in whom a large pelvic hydatid cyst and multiple hepatic hydatids were diagnosed by ultrasonography. The large pelvic hydatid cyst was drained through the posterior fornix under the guidance of ultrasound, and an external cephalic version was performed. A healthy baby was delivered vaginally with head presentation at term.

**Conclusion:**

HCD during pregnancy presents with management difficulty. It is important to formulate individualized treatment plans according to the actual situation of the patient and the local level of treatment.

**Supplementary Information:**

The online version contains supplementary material available at 10.1186/s12884-022-05180-2.

## Background

Hydatid cystic disease (HCD) is a zoonotic infection of sheep and cattle caused by the larval stage of the tapeworm *Echinococcus granulosus* (*E. granulosus*) [1]. Humans are infected by ingestion of food contaminated with the eggs of *E. granulosus* [1]. HCD is distributed worldwide and is more prevalent in rural areas with poor living conditions and poor sanitation facilities where sheep, dogs and humans live in close contact [2]. The Tibet Autonomous Region (TAR) of China is reported to be one of the most serious endemic regions for HCD worldwide [3]. Women are at higher risk of HCD than men in this area since women perform the major housework, including feeding dogs, grazing animals, milking and collecting cow dung, increasing their exposure to the eggs of *E. granulosus* [4]. Currently, free screening and treatment of hydatid disease are performed in local areas every year. However, due to the factors of personal health care awareness, lifestyle and geographical environment, hydatid disease remains a major public health problem in the TAR.

During pregnancy, the diagnosis of HCD is much more important because a decrease in cellular immunity can cause a rapid increase in parasitic growth, and the cysts enlarge [5]. HCD can cause some complications in pregnancy, which vary depending on the site and size of the cyst. Here, we report a case of a large pelvic hydatid cyst with breech presentation who was admitted to a secondary hospital at late pregnancy in the TAR.

## Case presentation

**Fig. 1:**
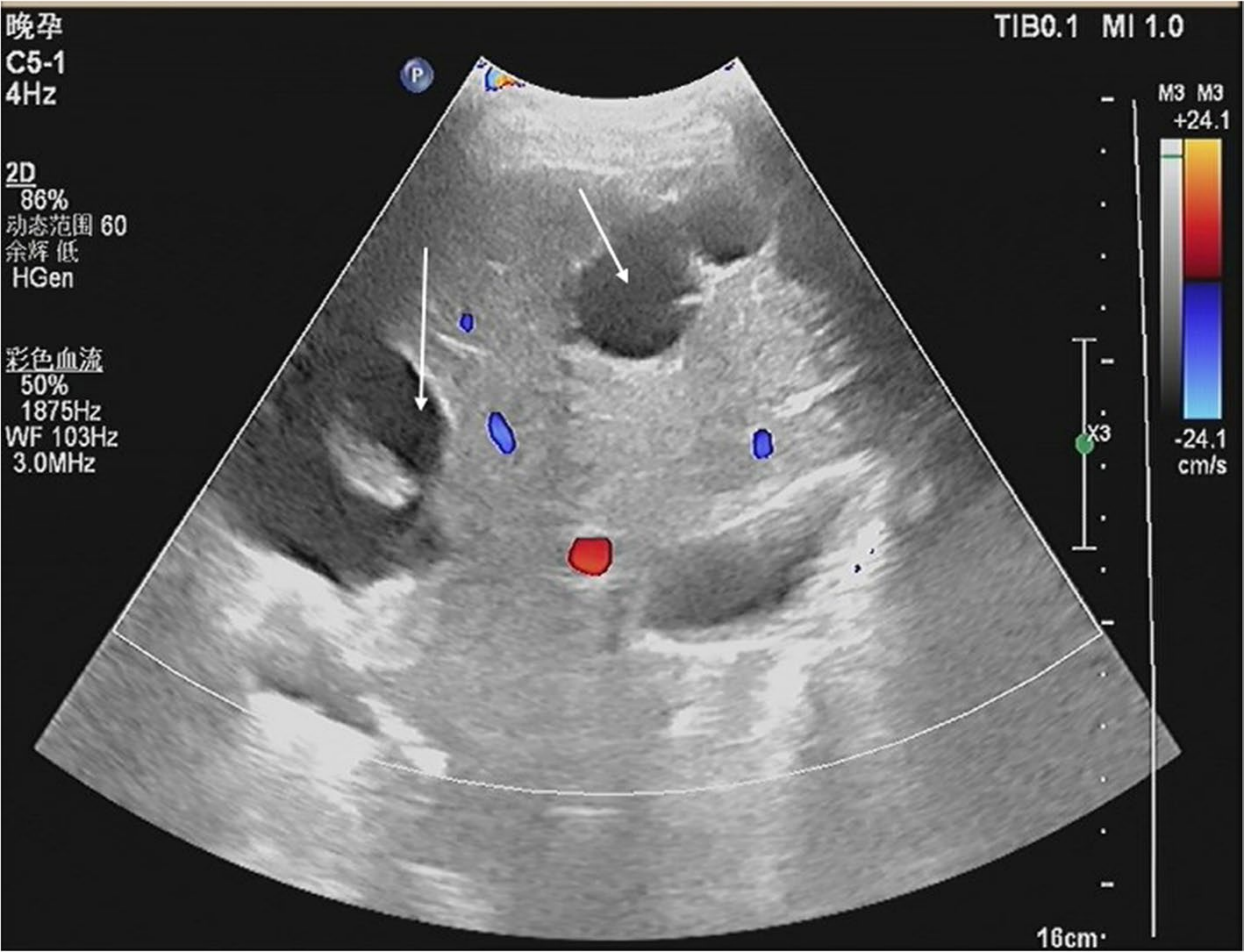
Sonography at 37 weeks of gestation. Multiple hepatic hydatids are shown (the largest cyst: 6.0*4.3 cm) (arrow)

**Fig. 2:**
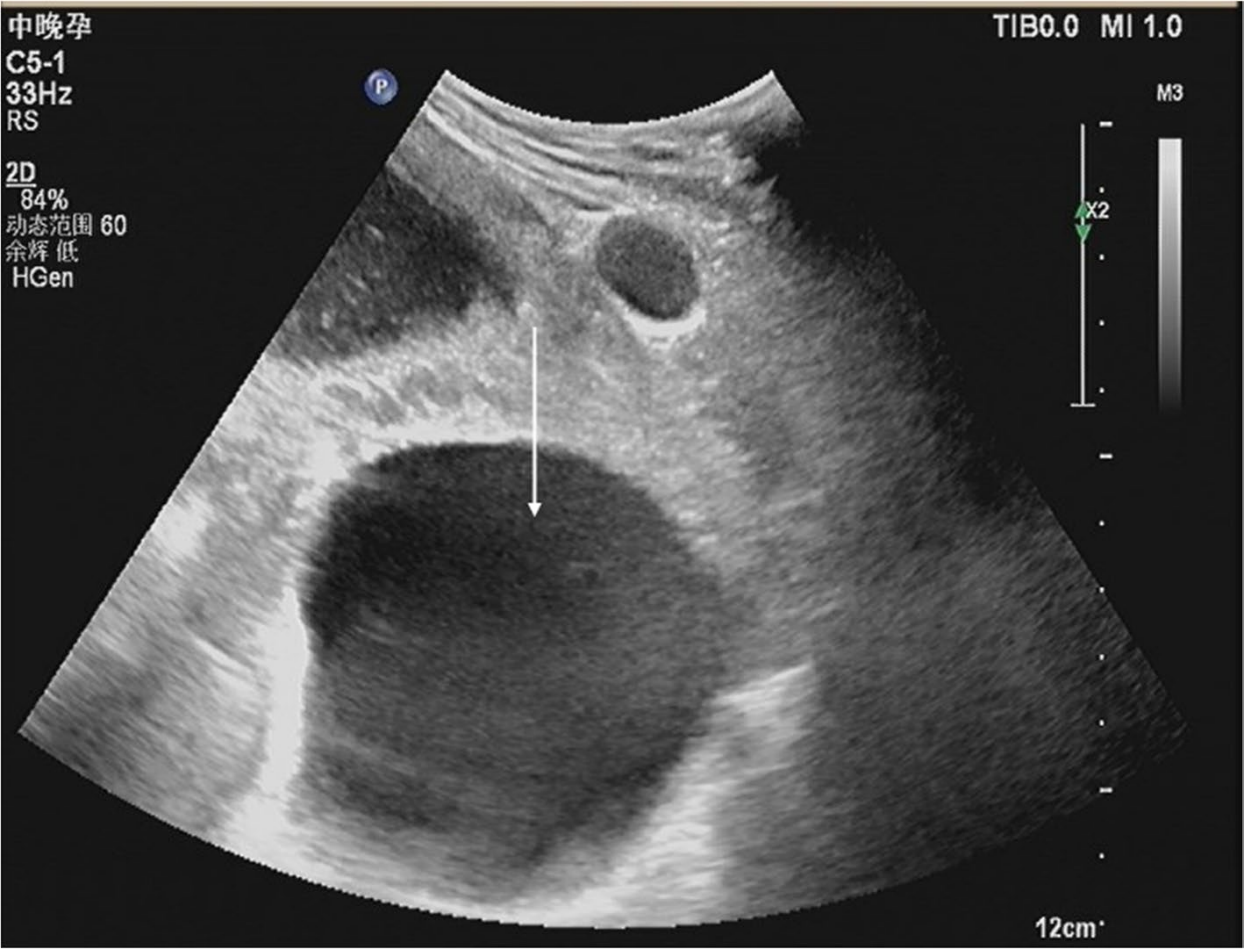
Sonography at 37 weeks of gestation. A large pelvic hydatid cyst is shown (10.0*8.2 cm) (arrow)

**Fig. 3:**
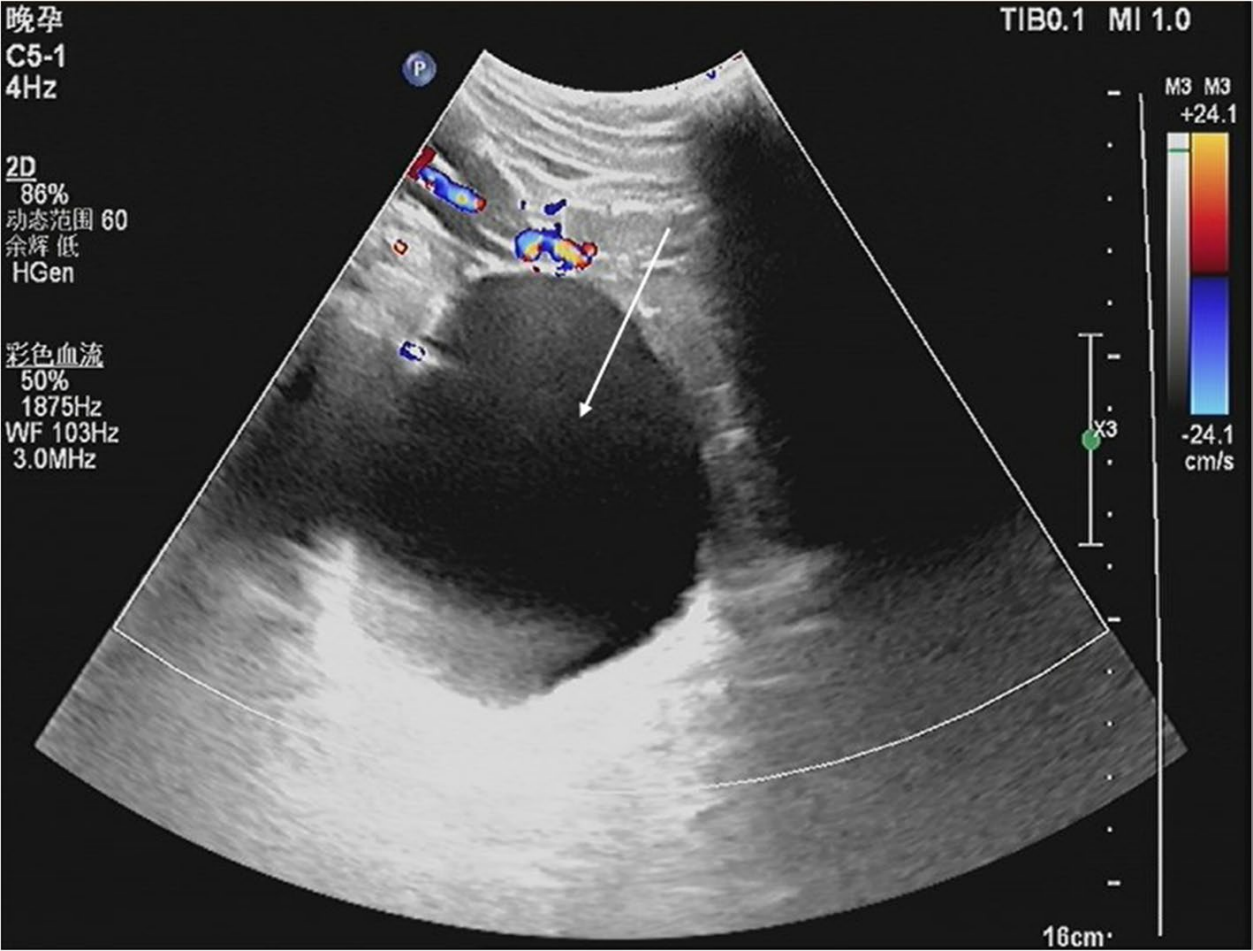
Sonography at 34 weeks of gestation. A large pelvic hydatid cyst is shown (9.5*8.6 cm) (arrow)

A 19-year-old woman, gravida 2, para 1, was admitted to a secondary hospital at 37 weeks of gestation with breech presentation. Her pregnancy was complicated by multiple hepatic hydatids (the largest cyst: 6.0*4.3 cm) (see Fig. 1) and a large pelvic hydatid cyst (10.0*8.2 cm) (see Fig. 2). Fetal ultrasound showed that the term of the fetus was approximately 37 weeks [biparietal diameter (BPD): 8.9 cm, abdominal circumference (AC): 31.7 cm, femur length (FL): 67 cm]. Three weeks prior to admission (September 1, 2021), the patient felt distended and presented at the hospital, and at that time, it was also her first antenatal examination during this pregnancy. An obstetric ultrasound was performed, and hepatic and pelvic hydatids were found during the scan (see Fig. 3). The size of the pelvic hydatid cyst did not change over these weeks (9.5*8.6 cm). Three years earlier, she delivered her first baby at home. Two years earlier, she was screened for local endemic diseases and found to have hepatic and abdominopelvic hydatid disease and underwent surgical treatment. Since then, due to a lack of conscientiousness for medical care and her living in remote pastoral areas, she did not have regular reexaminations or medication.

Ultrasound revealed that the pelvic hydatid cyst was located posterior to the uterus as a bulge in the posterior fornix (see Fig. 3). Vaginal and rectal examinations confirmed the mass to be posterior to the uterus and anterior to the rectum. Other laboratory tests, including routine blood examination, C-reactive protein, and liver and kidney function tests performed after admission, were normal.

**Fig. 4:**
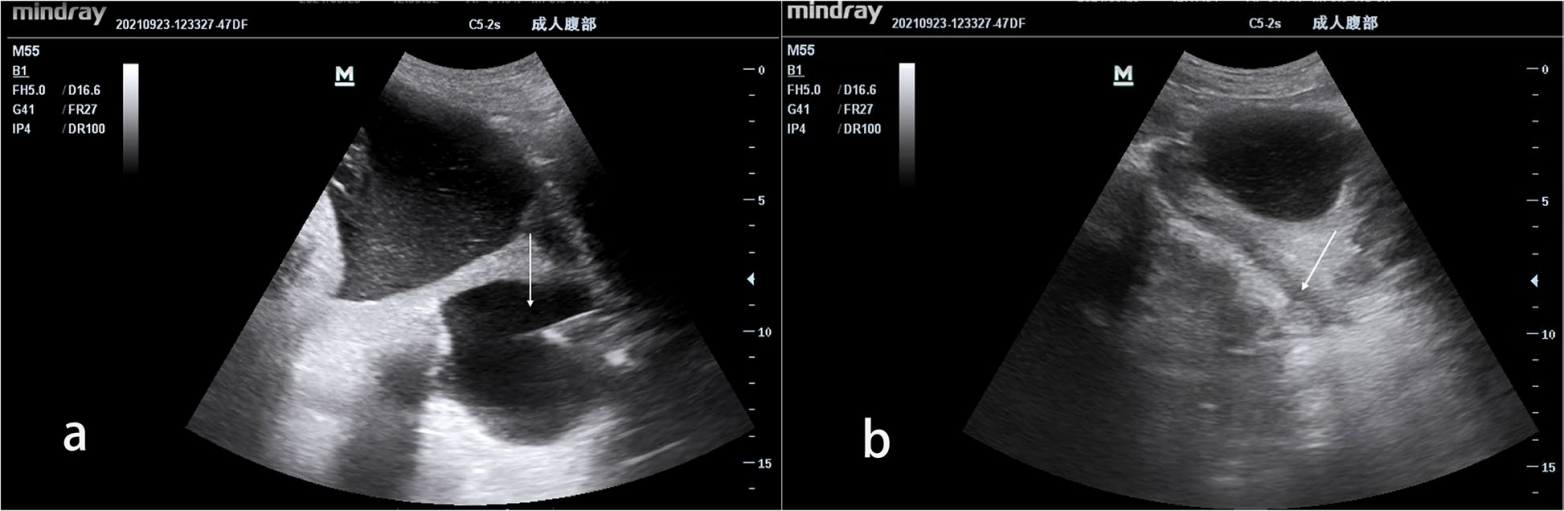
**a** Sonography at 37 weeks of gestation. It shows draining of the liquid of the cyst through the posterior fornix under the guidance of ultrasound (arrow). **b** Sonography at 37 weeks of gestation. After draining of the liquid of the pelvic hydatid cyst through the posterior fornix under the guidance of ultrasound, the cyst disappeared (arrow)

The woman declined Caesarean section and truly wanted natural delivery, so we devised a management plan according to her situation. Since a large cyst might lead to labor obstruction and abnormal fetal presentation and be more prone to rupture in late pregnancy, we chose to drain the cyst through the posterior fornix under the guidance of transabdominal ultrasound (see Fig. 4a and b; Video 1) (draining out approximately 400 ml of liquid). After the cyst disappeared, the patient was carefully observed for half of the day, including bleeding, fetal movement and uterine contraction. After confirming that the patient did not have any complications, we successfully performed an external cephalic version. After two days of observation, the woman was discharged because she showed no signs of labor. One week later, the woman delivered a baby boy naturally with head presentation in our hospital and was advised to undertake medical management for HCD follow-up.

## Discussion and conclusions

HCD in TAR is prevalent due to living habits, living in remote pastoral areas, traffic inconvenience, and lack of conscientiousness for medical care [6, 7]. Since these personal and environmental factors are unlikely to change in a short time, HCD has remained a major public health problem for local people for some time.

HCD can be located in the liver (63%), lungs (25%), muscles (5%), bones (3%), kidneys (2%), spleen (1%), and other sites (1%) [8]. It is sometimes nonsymptomatic and sometimes can cause complications, and these factors are closely related to the site and size of the cyst. The complications are mainly caused by compression, irritation or rupture of the cyst. If it is located in the liver, it can cause pain, vomiting, pruritus, jaundice and emergence of a mass [9]. If it is located in the cerebrum, it can commonly cause headache, vomiting, seizures and visual disturbances [10]. During pregnancy, if HCD is located in the pelvis, it can cause torsion, rupture, premature delivery, and labor obstruction [11, 12].

Many studies have suggested that ultrasound is the gold-standard screening test for HCD since it can show the number of cysts and their locations and relationships with other structures [9, 12]. Specific immunoglobulin determination can help to confirm the diagnosis. In addition, MRI is also a good examination method. However, advanced examination and medical facilities are relatively scarce in primary hospitals, and the cost is high. Therefore, ultrasound is also the most commonly used screening method for HCD in the TAR.

The ideal treatment for HCD has not been fully determined [13], and management during pregnancy is also a problem. Surgical therapy is a common approach, but it is difficult to perform during pregnancy since it can increase intraoperative morbidity due to the enlarged gravid uterus and pose a risk of miscarriage or preterm labor. Percutaneous treatment is a well-established procedure in HCD [14, 15], and it is also an effective treatment during pregnancy [16, 17]. However, there is a risk of anaphylaxis during the procedure [18]. For medical treatment, albendazole is a commonly used drug for echinococcosis [1]. It can reduce the size and number of cysts. The average time for a cyst to shrink or disappear using drugs is approximately 6–12 months [19, 20]. During pregnancy, the use of the drug remains controversial. It has been shown to have teratogenic effects in experimental studies [21]. In recent years, it has been reported that it is not absolutely contraindicated during pregnancy and can be used in the second and third trimesters if treatment is necessary [1]. Therefore, the management of HCD should be individualized. In the TAR, with the improvement of diagnosis and treatment in recent years, medical treatment of HCD is widely available, and surgical therapy can be administered in most tertiary hospitals and some secondary hospitals.

In this case, the patient truly hoped for vaginal delivery. However, the large pelvic hydatid cyst obstructed the birth canal, and the fetus was mispresented. The patient had been operated on for abdominal echinococcosis before, and the adhesion might have been serious. Hence, even if Caesarean section is performed, the difficulty might increase, especially in secondary hospitals, where the form of medical care provided to patients is affected by geographical location due to how long it takes to transfer patients, the nonavailability of blood banks and the lack of advanced medical facilities. Based on the location of the cyst, we modified the percutaneous treatment and opted to drain the cyst through the posterior fornix under the guidance of transabdominal ultrasound. There have been few reports of treatment adopting this procedure. After confirming that the patient did not have any complications, we performed an external cephalic version for the woman, and she finally had a successful vaginal delivery. This individualized treatment not only met the requirements of the patient but also caused minimal damage to the patient and was costless and effective.

In conclusion, HCD during pregnancy presents with management difficulty. It is important to formulate individualized treatment plans according to the actual situation of the patient and the local level of treatment.

## Supplementary Information

**Additional file 1: Video 1.** Draining the cyst through the posterior fornix under the guidance of transabdominal ultrasound.

## Data Availability

The data and materials of this study are not publicly available due to patient privacy and the hospital data management policy but are available from the corresponding author upon reasonable request. Such requests must also be approved by the local hospital.

## Abbreviations

HCD: hydatid cystic disease
TAR: Tibet Autonomous Region
BPD: biparietal diameter
AC: abdominal circumference
FL: femur length
